# Spatiotemporal diversity, structure and trophic guilds of insect assemblages in a semi-arid Sabkha ecosystem

**DOI:** 10.7717/peerj.860

**Published:** 2015-03-24

**Authors:** Haroun Chenchouni, Taha Menasria, Souad Neffar, Smail Chafaa, Lyès Bradai, Rachid Chaibi, Mohamed Nacer Mekahlia, Djamel Bendjoudi, Abdelkrim Si Bachir

**Affiliations:** 1 Department of Natural and Life Sciences, Faculty of Exact Sciences and Natural and Life Sciences, University of Tebessa, Tebessa, Algeria; 2 Department of Applied Biology, Faculty of Exact Sciences and Natural and Life Sciences, University of Tebessa, Tebessa, Algeria; 3 Department of Natural and Life Sciences, Faculty of Sciences, University of El Hadj Lakhdar, Batna, Algeria; 4 Department of Biology, Faculty of Natural and Life Sciences, University of Kasdi Merbah, Ouargla, Algeria; 5 Department of Biology, Faculty of Sciences, University of Amar Telidji, Laghouat, Algeria; 6 Department of Biology of Populations and Organisms, Faculty of Agro-veterinary and Biology, University of Saad Dahlab, Blida, Algeria

**Keywords:** Entomological biodiversity, Sabkha Djendli, Insect community ecology, Pitfall trapping, Algeria, Ecological niche, Functional groups, Conservation biology, Inland wetlands, Semi-arid lands

## Abstract

The current study highlights some knowledge on the diversity and structure of insect communities and trophic groups living in Sabkha Djendli (semi-arid area of Northeastern Algeria). The entomofauna was monthly sampled from March to November 2006 using pitfall traps at eight sites located at the vicinity of the Sabkha. Structural and diversity parameters (species richness, Shannon index, evenness) were measured for both insect orders and trophic guilds. The canonical correspondence analysis (CCA) was applied to determine how vegetation parameters (species richness and cover) influence spatial and seasonal fluctuations of insect assemblages. The catches totalled 434 insect individuals classified into 75 species, 62 genera, 31 families and 7 orders, of which Coleoptera and Hymenoptera were the most abundant and constant over seasons and study stations. Spring and autumn presented the highest values of diversity parameters. Individual-based Chao-1 species richness estimator indicated 126 species for the total individuals captured in the Sabkha. Based on catch abundances, the structure of functional trophic groups was predators (37.3%), saprophages (26.7%), phytophages (20.5%), polyphages (10.8%), coprophages (4.6%); whereas in terms of numbers of species, they can be classified as phytophages (40%), predators (25.3%), polyphages (13.3%), saprophages (12%), coprophages (9.3%). The CCA demonstrated that phytophages and saprophages as well as Coleoptera and Orthoptera were positively correlated with the two parameters of vegetation, especially in spring and summer. While the abundance of coprophages was positively correlated with species richness of plants, polyphage density was positively associated with vegetation cover. The insect community showed high taxonomic and functional diversity that is closely related to diversity and vegetation cover in different stations of the wetland and seasons.

## Introduction

Wetlands are recognized as important ecosystems in terms of biodiversity and functional role. These ecosystems include a remarkable range of habitats that are ecologically considered among the most productive ecosystems worldwide, with large socio-economic importance and high heritage values for humanity. They play crucial and major ecological functions, including trapping, absorbing and eliminating of potential toxic chemicals and pollutants, storage of natural carbon, recycling of nutrients, as well as they contribute to groundwater recharge in arid and semi-arid regions. Unfortunately, wetlands are experiencing rapid degradation due to severe transformations related to intensive human activities (Bobbink et al., 2006; Mitsch et al., 2009).

More than 2000 wetlands are listed in Algeria, including 50 sites classified on the Ramsar list of wetlands of international importance (Balla, 2012). Most of large inland saline depressions and backwaters “Sabkhas, Chotts, and Oases” are located in arid and semi-arid regions, with a unique agglomeration of this type of sites in northeastern of the country (Chenchouni & Si Bachir, 2010). The most characteristic type of the Algerian wetlands is seasonal/intermittent endorheic type that consists of Sabkha ecosystems “saline lakes” with typical alternation of drought phase in summer and flooding in winter (Khaznadar, Vogiatzakis & Griffiths, 2009; Balla, 2012).

Large-scale conservation programs focused on wetlands because these habitats support both terrestrial and aquatic biota where biodiversity therein is remarkably high (De Roeck et al., 2007). This biodiversity is the key factor maintaining the structure, stability, and functioning of these ecosystems (Ivask et al., 2008). What makes its conservation at different organizational levels (individual, population, community, ecosystem) has become an issue that deserves national and international attention (Bobbink et al., 2006; Montagna et al., 2012). Moreover, regional contributions have also proven their impact in improving the knowledge and conservation of these habitats (Piñero et al., 2011; Chaibi et al., 2012; Guezoul et al., 2013).

As a biological model, invertebrates embrace a large species richness ranging over several taxa with large magnitude of sizes. They colonise various microhabitats and perform an extraordinary diverse functional roles, constituting thus key organisms at different trophic levels inside food webs of wetland ecosystems (Koricheva et al., 2000; Finke & Denno, 2002; Haddad et al., 2009; Piñero et al., 2011). Although they are of relevant importance in the ecosystem functioning of wetlands, invertebrates were slightly used as criteria in conservation programs of wetlands compared to specific criteria based on waterbirds and fishes, since only recently these organisms as well as other taxa were included in the ninth criterion used by Ramsar Convention for considering wetlands internationally important (Mitsch et al., 2009; Chenchouni & Si Bachir, 2010).

Furthermore, it is well known that biodiversity and structure of invertebrates, particularly insects, in saline inland temporary wetlands are governed by two main abiotic factors: hydroperiod “water regimes” and salinity (Bilton, Freeland & Okamura, 2001; Brock, Nielsen & Crossle, 2005; Gascon et al., 2005; Waterkeyn et al., 2008), whereas the involved biotic factors are dealing with vegetation traits and various biotic interactions of food webs (Koricheva et al., 2000; Carver et al., 2009; Haddad et al., 2009). However, although species diversity is a good parameter for valuing structure of invertebrate communities and defining conservation strategies, scarcity of species should also be taken into account (Nijboer & Verdonschot, 2004).

The multi-scale ecological surveys that investigated animal biodiversity of the Algerian wetlands, specifically at the northeast of the country, they focused on waterbirds (e.g., Samraoui & Samraoui, 2008), fishes (e.g., Chaibi et al., 2012), and some other taxa like dragonflies (e.g., Samraoui et al., 2011), whereas the ecology of terrestrial arthropods of Sabkha ecosystems remain very little studied in these saline environments (Hogarth & Tigar, 2002).

Located in high plains of Northeast Algeria, the Sabkha Djendli is a seasonal salt lake whose flora was thoroughly surveyed throughout the waterbody vicinity (Neffar, Chenchouni & Si Bachir, in press). However, there had been very little investigation of faunal communities, including insects, inhabiting the Sabkha and its environs in connection with their biotope, except for some ornithological surveys of wintering waterbirds (e.g., Samraoui & Samraoui, 2008; Bensizerara et al., 2013).

The study of relationships between spatiotemporal variation of invertebrate communities and ecological parameters provides valuable information for conservation assessment and restoration planning, and may efficiently guide the implementation of future management program (Comin & Comin, 1992; Fischer & Lindenmayer, 2007; Montagna et al., 2012). Furthermore, the assessment of functional trophic groups is crucial to outlining the structure of food webs and, accordingly, identifying any perturbation in the ecosystem functioning (Chesson & Huntly, 1997; Gascon et al., 2005), particularly under changing environmental conditions. Indeed, some insect groups such as dragonflies, hoverflies and some ground beetles (particularly Carabidae) represent good indicators of biodiversity assessment and monitoring in wetlands and mesic environments (Rainio & Niemelä, 2003; Sánchez-Fernández et al., 2006; Hepp & Melo, 2013). In fact, the core aim of the current study is placed within the perspective of insect biodiversity assessment for conservation purpose as outlined here above.

Thereby, the objectives of this pioneer study are dealing with the framework of understanding the entomofauna composition of Sabkha Djendli. This treatise aims to (i) provide accurate information on the spatiotemporal variation of the composition, structure and diversity indices of the insect community inhabiting the vicinity of the Sabkha; (ii) evaluate ecological status and diversity of the functional trophic groups in relation to seasons and site orientations of the salt lake; (iii) understand the structural and functional similarities of insect communities living around the Sabkha; (iv) assess the effect of seasons and site orientations on the spatiotemporal abundance variations of both insect orders and functional trophic groups; and (v) determine how climate and vegetation parameters influence spatial and seasonal fluctuations of insect assemblages.

## Materials and Methods

### Study area

**Figure 1:**
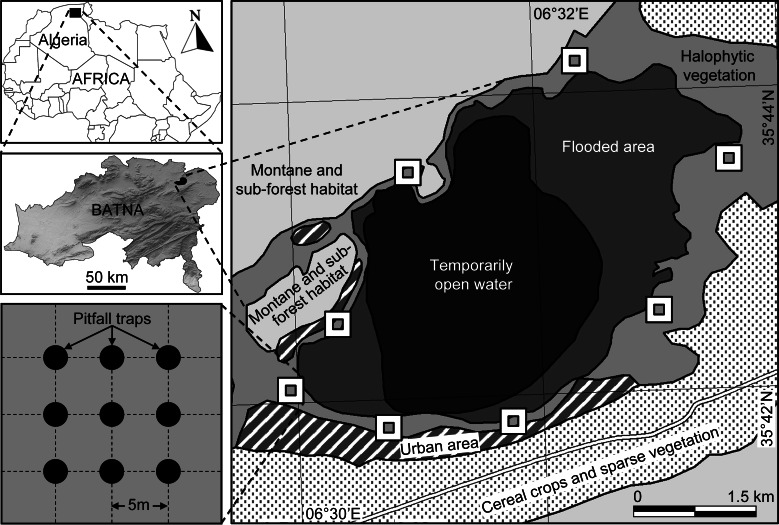
Location and sampling design. Location of the sampled station and sites at Sabkha Djendli (Batna, Northeast Algeria) and sampling design of pitfall traps.

Sabkha Djendli (35°42′56″N, 6°31′46″E) belongs to the eco-complex of wetlands located in the High Plains “Hauts Plateaux” region in eastern Algeria (Fig. 1). The site is a temporary lake with brackish/salt water that highly depends on rainfall amounts and water regime. Sabkha Djendli covers about 3,700 ha with an average altitude of 833 m in an area where inhabitants are mainly involved in agricultural activities like cereal and fruit cultivation and livestock of sheep and cattle. The adjacent land use types of this area includes scattered and low intensity urban use within extended rainfed cereal crops of barley, durum and bread wheat. The nearby mountains are covered by open shrub-forest vegetation, where the main tree species are *Juniperus phoenicea*, *Quercus ilex* and *Olea oleaster* (Bensizerara et al., 2013).

Based on meteorological data provided by the meteorological station of Batna (WMO Id: 60468) of the period 1974–2014, the climate of the study area is typically semi-arid Mediterranean, characterised by cold-wet winters and hot-dry summers. The dry period extends over four months from June to September. Precipitation is erratic and there are large temporal variations. The coldest month is January with an average temperature of 5.3 °C, and the hottest month is July with an average temperature of 25 °C. The relative humidity of the air fluctuates between 40% and 75% and the winds are generally low in dominance west to south-west, with the passage of Sirocco in summer during July–August.

The natural vegetation is represented by halophytes such as *Atriplex halimus*, *Suaeda fructicosa*, *Suaeda vermiculata*, and *Sarcocornia fructicosa*, but also other spontaneous vegetation like *Tamarix gallica*, *Artemisia herba-alba* and *Juncus maritimus* (Neffar, Chenchouni & Si Bachir, in press). The current entomological survey was carried on the belt of halophytic vegetation surrounding the Sabkha (Fig. 1).

### Sampling design

At eight cardinal and inter-cardinal points of the site border of Sabkha Djendli, the insect fauna was monthly sampled during the period March to November 2006. Halophytic vegetation dominated in the entire sampled area. At each sampling points, insects were trapped using nine pitfall traps (Spence & Niemelä, 1994), which were set up inside a square plot of 400 m^2^ (20 m × 20 m). These uncovered traps are aligned 3–3 along three rows and spaced from each other with 5 m (Fig. 1). Each trap was filled to 3/4 of water containing a wetting agent, and its catches were monthly recovered after one week trapping since first setting day. The caught specimens were identified by genus and species. The nomenclature and taxonomy of species were based on up-to dated references (Bouchard et al., 2011; de Jong, 2013; Ligeiro & Smetana, 2013; Anichtchenko et al., 2014; AntWeb, 2014; Eades et al., 2014).

### Data mining

Data of insect catches from the nine uncovered traps were pooled to form one sample per sampling station per month. Data were presented by taxonomic orders and trophic groups and were expressed for orientation points and seasons (Table S1) to facilitate spatiotemporal comparisons for all the following parameters. The relative abundance (*RA*) was determined as the ratio of number of individuals rounded to the total number of individuals recorded (*Ni*). Occurrence frequency (Occ) was calculated for each species by the number of stations wherein the species was found / the total number of sampled stations (Magurran, 2004). Four species groups are distinguished by Bigot & Bodot (1973), according to their frequencies of occurrence: Very accidental species (Vac): an occurrence of less than 10%; Accidental species (Acc) occurrence varies between 10 and 24%; Common species (Cmt) are present in 25–49%; Constant species (Cst) are present in 50% or more of the samples.

### Species richness estimation

Biodiversity of insects was assessed by species richness observed “*S*_obs_,” which corresponds to the total number of identified insect species at each station or season. In addition, Shannon’s index (*H*′ = − ∑*pi* × log_2_*pi*) and evenness (Evenness =*H*′/log_2_*S*_obs_) were applied for measuring insect diversity in each sampled station and season period based on the relative density *pi* of the *i* th species (Magurran, 2004).

Estimated species richness was calculated with the program EstimateS 9.1.0 (Colwell, 2013). *S*_est_ was extrapolated by the selection of the least biased and most precise estimator (Brose & Martinez, 2004). We applied the following nonparametric species richness estimators: (i) *S*_est_ (analytical) with lower and upper bounds of 95% Confidence Interval ‘CI,’ which gives the expected number of species represented among a given number of individuals (Colwell et al., 2012), (ii) Chao 1 richness estimator with log-linear 95% confidence interval lower and upper bounds (Chao, 1984). These two estimators were selected because of the involved assumptions about the underlying species abundance distribution remain fewer. However, instead of using the bias-corrected form of the Chao-1 estimator, we chose the larger of Chao-1 Classic and ACE ‘Abundance-base Coverage Estimator’ because the coefficient of variation (CV) of the abundance in our dataset was high (i.e., CV >0.5). Number of singletons (species with only one individual among total individuals) and doubletons (species with only two individuals among total individuals) were given as mean (±standard deviation ‘SD’) among 100 runs of randomizations. Detailed descriptions of these estimators and procedures can be found in Colwell (2013). In order to assess the diversity of the entomofauna of Sabkha Djendli as a whole, interpolated species accumulation curves “individual-based rarefaction” (Magurran, 2004; Colwell et al., 2012) of the total data were computed with both previous estimators (*S*_est_ and Chao-1 with 95% CI). Model of total raw data is multiple individual-based abundance samples (batch input, including stations and seasons: Table S2) applied for species richness estimation using EstimateS 9 (Colwell, 2013). Rarefaction curves were made by repeatedly sampling the collected species with 100 randomisations of individual orders without replacement. With the plot of individual-based rarefaction curves, we incorporated the means of singletons and doubletons (±SD) in order to allow comparison of species richness.

Using the Bray-Curtis index (= Sørensen quantitative index), similarity indices were computed between stations taken in pairs. The obtained proximity matrix allows comparing species richness, while the Bray-Curtis index takes observed abundances into account (Magurran, 2004). For the estimated data, Chao’s abundance-based Jaccard index (Chao et al., 2005) was used to compare insect species richness between study stations taken in pairs. We computed the raw Chao Abundance-based Jaccard index (not corrected for undersampling bias) as well as the estimators of their true values, so the effect of the bias correction on the index can be assessed between stations. The model of raw data used for the analysis using EstimateS 9.1.0 was Format 1 of sample-based abundance data “Filetype 1” (Table S3). The free software EstimateS (Colwell, 2013) was used in computation of all species richness and diversity indices.

### Statistical analyses

Agglomerative hierarchical clustering (AHC) was applied to cluster sampled stations according to their species abundances based on the proximity matrix including values of Bray-Curtis index. The agglomeration method we used was the unweighting pair-group average.

Moreover, Pearson’s Chi-squared test (*χ*^2^) was applied to look for dependencies between the distributions of structural traits values (*Ni*, *S*_obs_, *Ni*/*S*_obs_, *H*′, Evenness) of the functional trophic groups among both study stations and seasons.

Generalized linear models (GLMs) were applied to test spatiotemporal variations of abundances of both taxonomic orders and trophic groups following the effects of ‘Orientation,’ ‘Season’ and their interaction ‘Orientation × Season.’ As all abundances were count data, GLMs were fitted using a Poisson distribution error and log link function (Myers et al., 2012). Computations were carried out with the help of R (R Core Team, 2015) using the ‘glm’ function and ‘AIC’ function to calculate the Akaike’s information criterion (AIC) as model simplification. Likelihood-ratio tests “LR” were performed for each GLM to assess the effects of explicative factors (Orientation, Season and Orientation × Season). Each “LR” was tested using sequential “Type-I” under the ‘Anova’ function that computes the deviance (*χ*^2^) and the corresponding *P*-value (Fox, 2008).

The spatiotemporal gradients of insect assemblages were analyzed in relation with vegetation traits using a canonical correspondence analysis (CCA). The data used were the abundances of both taxonomic orders and trophic groups on the study seasons and orientations where they were counted. For the spontaneous vegetation, two parameters were assessed at each orientation and season: the vegetation cover (%) and total species richness (number of plant species). These data were generated from Neffar, Chenchouni & Si Bachir (in press). Since the CCA has the ability to combine ordination and gradient analysis functions in a readily interpretable manner, it was applied to relate spatiotemporal insect abundances to vegetation variables in order to highlight relationships between spatiotemporal variations of insects and vegetation traits as explanatory variables (Jongman, Ter Braak & Tongeren, 1995). At the end of overcoming the disadvantage effect of scale differences in data, insect densities as well as vegetation variables were normalized using normal distribution transformation based on the average and standard deviation of each input.

Finally, Pearson’s correlation was used to test the significance of relationships between densities of insect assemblages (of both taxonomic orders and trophic groups) and vegetation parameters (vegetation cover and species richness) and some climate parameters originated from Batna weather station of the year 2006 (Table S4). These parameters are mean temperature (°C), precipitation amount (mm), mean humidity (%), mean wind speed (Km/h) and number of rain days. The climatic parameters considered were computed for each season as average/sum of the daily data of 45 days prior the trapping period. This period duration is estimated to be appropriate for affection both the biological cycle and population dynamics of most insects of the region (Chafaa et al., 2013; Idder-Ighili et al., in press). All correlations were carried out as pairwise two-sided tests using the function ‘rcorr.adjust’ in R (R Core Team, 2015).

## Results

### Taxonomic composition of insect community

Pitfall sampling of entomofauna at Sabkha Djendli revealed an insect community composed of 75 species from 434 individuals caught. This entomofauna can be classified into 7 orders, 31 families and 62 genera (Table 1). Coleoptera was the best represented with 238 (54.8%) individuals caught belonging to 39 species and 15 families, followed by Hymenoptera with 149 (34.3%) individuals of 18 species and 8 families, then came Orthoptera with 22 individuals (10 species and 2 families). The orders Dermaptera, Heteroptera, Homoptera and Diptera were poorly represented by either species or catch abundance. Furthermore, the identified entomofauna included five functional trophic groups: phytophages with 30 species, predators with 19 species, polyphages with 10 insects, saprophages with 9 species and coprophages with 7 species.

**Table 1:** Insect listing. Systematic list, trophic status, abundances and occurrences of insect species captured using pitfall traps at edges of Sabkha Djendli, Northeast Algeria.

Classification (RA in %)	Species	FTG	Ni	RA	Occ	Scale
O: DERMAPTERA (3.2)						
F: Forficulidae (3.2)	*Anisolabis mauritanicus*	Pol	4	0.92	16.7	Acc
	*Forficula auricularia*	Pol	10	2.30	25.0	Cmt
O: ORTHOPTERA (5.1)						
F: Gryllidae (2.1)	*Acheta domesticus*	Phy	3	0.69	12.5	Acc
	*Gryllus bimaculatus*	Phy	2	0.46	8.3	Vac
	*Gryllus campestris*	Phy	2	0.46	8.3	Vac
	*Gryllus* sp.	Phy	2	0.46	8.3	Vac
F: Acrididae (3.0)	*Acrotylus patruelis*	Phy	3	0.69	12.5	Acc
	*Calliptamus barbarus*	Phy	4	0.92	12.5	Acc
	*Ephippiger* sp.	Phy	1	0.23	4.2	Vac
	*Oedipoda fuscocincta*	Phy	3	0.69	12.5	Acc
	*Sphingonotus rubescens*	Phy	1	0.23	4.2	Vac
	*Sphingonotus* sp.	Phy	1	0.23	4.2	Vac
O: HETEROPTERA (0.2)						
F: Lygaeidae (0.2)	*Lygaeus sexatilis*	Phy	1	0.23	4.2	Vac
O: HOMOPTERA (0.2)						
F: Cicadellidae (0.2)	*Cicadela variabilis*	Phy	1	0.23	4.2	Vac
O: COLEOPTERA (54.8)						
F: Cicindelidae (0.7)	*Calomera littoralis*	Pre	1	0.23	4.2	Vac
	*Cassolaia maura*	Pre	2	0.46	8.3	Vac
F: Callistidae (21.9)	*Calathus circumseptus*	Sap	95	21.89	87.5	Cst
F: Carabidae (12.2)	*Calathus* sp.	Pre	1	0.23	4.2	Vac
	*Macrothorax morbillosus*	Pre	1	0.23	4.2	Vac
	*Carabus* sp.	Pre	13	3.00	37.5	Cmt
	*Scarites laevigatus*	Pre	16	3.69	41.7	Cmt
	*Scarites* sp.	Pre	5	1.15	16.7	Acc
	*Zabrus* sp.	Phy	17	3.92	33.3	Cmt
F: Geotrupidae (0.9)	*Geotrupes*sp.	Sap	4	0.92	16.7	Acc
F: Scarabaeidae (9.2)	*Geotrogus* sp.	Sap	7	1.61	16.7	Acc
	*Anomala dubia*	Pol	17	3.92	20.8	Acc
	*Bubas bison*	Cop	4	0.92	16.7	Acc
	*Gymnopleurus flagellatus*	Cop	4	0.92	16.7	Acc
	*Onthophagus taurus*	Cop	5	1.15	20.8	Acc
	*Oxythyrea funesta*	Phy	1	0.23	4.2	Vac
	*Scarabaeus sacer*	Cop	1	0.23	4.2	Vac
	*Scarabaeus* sp.	Cop	1	0.23	4,2	Vac
F: Silphidae (0.2)	*Silpha opaca*	Pre	1	0.23	4.2	Vac
F: Staphylinidae (0.7)	*Staphylinus olens*	Pol	3	0.69	12.5	Acc
F: Cetonidae (0.5)	*Cetonia ablonga*	Phy	1	0.23	4.2	Vac
	*Cetonia funeraria*	Phy	1	0.23	4.2	Vac
F: Cantharidae (0.2)	*Cantharis* sp.	Phy	1	0.23	4.2	Vac
F: Meloidae (1.4)	*Mylabris crocata*	Phy	1	0.23	4.2	Vac
	*Mylabris quadripunctata*	Phy	2	0.46	8.3	Vac
	*Mylabris variabilis*	Phy	3	0.69	12.5	Acc
F: Tenebrionidae (2.3)	*Adesmia microcephala*	Sap	1	0.23	4.2	Vac
	*Blaps mortisaga*	Sap	1	0.23	4.2	Vac
	*Blaps nitens*	Sap	1	0.23	4.2	Vac
	*Opatrum* sp.	Sap	2	0.46	8.3	Vac
	*Tentyria bipunctata*	Sap	1	0.23	4.2	Vac
	*Tentyria* sp.	Sap	4	0.92	12.5	Acc
F: Dermestidae (1.2)	*Dermestes* sp.	Cop	1	0.23	4.2	Vac
	*Trogoderma* sp.	Cop	4	0.92	16.7	Acc
F: Cucujidae (0.7)	*Canthartus* sp.	Pol	3	0.69	8.3	Vac
F: Curculionidae (0.5)	*Coniocleonus excoriatus*	Phy	1	0.23	4.2	Vac
	*Lixus punctiventris*	Phy	1	0.23	4.2	Vac
F: Chrysomelidae (2.3)	*Chrysomela* sp.	Phy	9	2.07	16.7	Acc
	*Entomoscelis* sp.	Phy	1	0.23	4.2	Vac
O: HYMENOPTERA (34.3)						
F: Formicidae (25.6)	*Camponotus* sp.	Pre	1	0.23	4.2	Vac
	*Cataglyphis bicolor*	Pre	67	15.44	58.3	Cst
	*Messor barbarous*	Pre	7	1.61	12.5	Acc
	*Pheidole pallidula*	Pol	1	0.23	4.2	Vac
	*Tapinoma nigerrimum*	Pre	9	2.07	20.8	Acc
	*Tetramorium biskrense*	Pre	26	5.99	50.0	Cst
F: Vespidae (0.2)	*Polistes gallicus*	Pre	1	0.23	4.2	Vac
F: Apidae (4.9)	*Apis mellifera*	Phy	10	2.30	29.2	Cmt
	*Apis* sp.	Phy	5	1.15	16.7	Acc
	*Bombus pascuorum*	Phy	2	0.46	4.2	Vac
F:Megachilidae (1.2)	*Megachile* sp.	Phy	5	1.15	16.7	Acc
F:Halictidae (0.9)	*Sphecodes* sp.	Phy	1	0.23	4.2	Vac
	*Halictus* sp.	Phy	3	0.69	12.5	Acc
F: Scoliidae (1.6)	*Scolia* sp.	Pre	7	1.61	25.0	Cmt
F: Sphecidae (0.7)	*Ammophila hirsute*	Pre	1	0.23	4.2	Vac
	*Ammophila sabulosa*	Pre	1	0.23	4.2	Vac
	*Sphex funerarius*	Pre	1	0.23	4.2	Vac
F: Mutillidae (0.2)	*Mutilla* sp.	Pre	1	0.23	4.2	Vac
O: DIPTERA (2.1)						
F: Tabanidae (0.5)	*Tabanus* sp.	Pol	2	0.46	8.3	Vac
F: Muscidae (0.7)	*Musca domestica*	Pol	1	0.23	4.2	Vac
	*Musca* sp.	Pol	2	0.46	8.3	Vac
F: Sarcophagidae (0.9)	*Sarcophaga* sp.	Pol	4	0.92	16.7	Acc

**Notes.**

RA relative abundance (%) FTG functional trophic groups Ni total number of caught individuals Occ occurrence frequency Cop coprophages Phy phytophages Pol polyphages Pre predators Sap Saprophages Vac very accidental species Acc accidental species Cmt common species Cst constant species

### Relative abundance and occurrence

The main species with high relative abundance (*RA*) of catch were *Calathus circumseptus* (21.9%), *Cataglyphis biskrense* (15.4%), *Tetramorium biskrensis* (6%), *Zabrus* sp. (3.9%), *Anomala dubia* (3.9%), *Scarites laevigatus* (3.7%) and *Carabus* sp. (3%), respectively. Furthermore, families that dominated in terms of catches belonged to Coleoptera and Hymenoptera, including Formicidae with a total of 111 individuals (25.6%), Callistidae with 95 individuals (21.9%), Carabidae with 53 individuals (12.2%) and Scarabeidae with 40 individuals (9.2%), and Apidae with 29 individuals the equivalent of 6.0% of total caches (Table 1).

Regarding spatial occurrence of insect species at the eight sampled stations, almost all species (66 species) were accidental and very accidental. Nevertheless, three species were constant (Occ ≥50%) during the study period: *Chlaenius circumseptus* (Callistidae), *Cataglyphis bicolor* (Formicidae) and *Tetramorium biskrensis* (Formicidae). Common species (Occ = 25–50%) were characterized by six species: *Scolia* sp. (Scoliidae), *Apis mellifera* (Apidae) *Zabrus* sp. (Carabidae) *Carabus* sp. (Carabidae) *Scarites laevigatus* (Carabidae) *Forficula auricularia* (Forficulidae) (Table 1).

### Spatiotemporal composition and diversity

The sampled station located southern Sabkha Djendli possessed the highest values of catch seize (93 individuals, *RA* = 21.4%), species richness (27 species) and the ratio *Ni*/*S*_obs_(3.4), whereas the highest values of Shannon index and evenness were respectively recorded at station of West, Southeast, South, and East. However, this later station (East) had the lowest values of insect composition (*Ni* = 43, *RA* = 9.9%, *S*_obs_ = 19).

As for seasons, values of diversity parameters of insect assemblages were higher during spring and autumn, with a slight leaning to spring values. However, the summer scored the lowest values. Overall, sampling insects using pitfall traps at Sabkha Djendli revealed a diversity equals to 4.7 according to Shannon index with an evenness of 0.76 (Table 2).

**Table 2:** Spatiotemporal diversity. Spatial and seasonal variation of the diversity parameters of insect assemblages in Sabkha Djendli, Northeast Algeria.

Parameters	Orientation								Season			Total
	S	SW	W	NW	N	NE	E	SE	Spring	Summer	Fall	
Abundances (*Ni*)	93	48	60	44	52	50	43	45	163	116	155	434
*RA* (%)	21.4	11.0	13.8	10.1	11.8	11.5	9.9	10.3	37.5	26.7	35.6	100
Species richness (*S*_obs_)	27	24	27	19	17	22	19	22	46	38	51	75
Ratio *Ni*/*S*_obs_	3.4	2.0	2.2	2.3	3.1	2.3	2.3	2.0	3.5	3.1	3.0	5.8
Shannon index (*H*′)	3.9	3.6	4.1	3.5	3.1	3.8	3.7	3.9	4.5	4.0	4.6	4.7
Evenness	0.81	0.79	0.86	0.83	0.77	0.84	0.86	0.87	0.82	0.76	0.82	0.76

The generalized linear models revealed different effects of the two factors ‘station orientation’ and ‘Season’ among abundances of insect taxonomic orders (Table 3). In general, the number of individuals of all orders combined significantly differed between stations (*P* < 0.001) and seasons (*P* = 0.011). The orientation of stations showed a significant effect on the variation of abundances of Dermaptera, Coleoptera and Hymenoptera. Only Hymenoptera varied significantly between seasons (*P* < 0.001), whereas the effect of the interaction ‘Orientation × Season’ was deemed significant for both Coleoptera (*P* = 0.006) and Hymenoptera (*P* < 0.001). The Akaike’s information criterion indicated that the GLM applied for Dermaptera abundances was the best-fitted model (AIC = 368).

**Table 3:** Abundances of insect orders. GLMs testing the effects of ‘Station orientation,’ ‘Season’ and their interaction on the variation of insect orders abundances in Sabkha Djendli, Northeast Algeria.

Variation	*Df*	*χ* ^2^	*P*	*χ* ^2^	*P*
		Dermaptera (AIC = 368)		Orthoptera (AIC = 880)	
Orientation	7	14.29	0.046	4.18	0.759
Season	2	0.55	0.760	2.52	0.283
Orientation × Season	14	16.89	0.262	21.40	0.092
		Heteroptera (AIC = 550)		Homoptera (AIC = 650)	
Orientation	7	4.16	0.761	4.16	0.761
Season	2	2.20	0.333	2.20	0.333
Orientation × Season	14	0.00	1.000	0.00	1.000
		Coleoptera (AIC = 1144)		Hymenoptera (AIC = 7118)	
Orientation	7	20.42	0.005	49.65	<0.001
Season	2	2.96	0.227	18.85	<0.001
Orientation × Season	14	30.93	0.006	77.15	<0.001
		Diptera (AIC = 463)		All orders (AIC = 13160)	
Orientation	7	7.24	0.404	29.72	<0.001
Season	2	2.91	0.233	9.05	0.011
Orientation × Season	14	13.04	0.523	20.58	0.113

### Estimates of species richness

**Figure 2:**
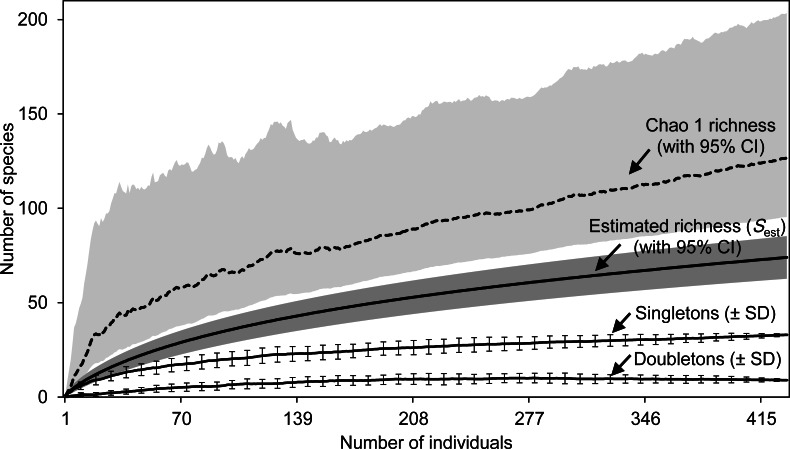
Estimated species richness. Species richness estimates (with 95% confidence intervals) for the estimators *S*_est_ (analytical) and Chao 1 Classic (dashed line) based on 100 randomized samples (Colwell, 2013) for the total data of entomofauna sampled in Sabkha Djendli, Northeast Algeria. Rarefaction curves were represented by means of singletons and doubletons (± standard deviations, only every 8th data point is shown).

For the whole Sabkha Djendli, the rarefaction curves kept increasing with the increase of number of individuals (Fig. 2). Expected species richness curves computed using the first-order Chao richness estimator proved to highly increase with the number of individuals captured all over the Sabkha. This estimator indicated that at a total of 434 individuals, Sabkha Djendli had 128 species (lower 95% CI: 96 species, upper 95% CI: 204 species). Chao-1 estimated species richness was significantly greater than the analytical estimated richness that indicated 75 species (upper 95% CI: 86 species). The individual-based rarefaction curve of singletons was higher than that of doubletons but both kept increasing to reach a plateau in all stations considered, suggesting that the expected species richness estimated with both previous estimators (*S*_est_ and Chao-1) increased with the increase of singletons rather than doubletons. At a total of 433 individuals, the mean of singletons was 32.97 ± 0.39 species, whereas doubletons provided an estimate of 9 ± 0.35 species (see Table S5 for all diversity statistics including other species richness estimators and species diversity indices computed for the total captures of insects at Sabkha Djendli).

### Spatial similarities of the entomofauna

The assessment of similarities of insect assemblages between the sampled stations revealed low similarities ranging between 0.161 and 0.581, based on Bray-Curtis index that involved insect abundances in comparisons. The highest values of that index were observed between North and East stations (Table 4). Most of the similarities did not exceed 0.500, except for the NE station with East (Bray-Curtis = 0.581) and SE (Bray-Curtis = 0.505) stations. The raw Chao’s abundance-based Jaccard index indicated similarities between rest of the stations ranged from 0.236 to 0.603. The high values of that index (>0.5) were recorded between south station and all the rest of the stations except SW. As for the estimated Chao’s abundance-based Jaccard index, all estimated similarities between stations were higher than 0.500 excluding the similarity between NW and West stations where it was 0.363. The program EstimateS provided other shared species statistics (Table S6).

**Table 4:** Spatial abundance-based similarities. Similarity matrix of observed (above the diagonal) and estimated (under the diagonal) insect assemblages in the studied sites at Sabkha Djendli, Northeast Algeria. Values of observed data (above the diagonal) are referred to Bray-Curtis index (and number of shared Species). The values of raw Chao’s abundance-based Jaccard index and the estimators of their true values (Chao et al., 2005), in brackets, are given under the diagonal. Station locations ‘orientations’ of the first row are associated with values of observed species richness (*S*_obs_), whereas orientations in the first column are followed by means of Chao-1 richness estimator and 95% CI indicated in square brackets.

Orientations	S (27)	SW (24)	W (27)	NW (19)	N (17)	NE (22)	E (19)	SE (22)
S (56.0)		0.414	0.355	0.412	0.472	0.479	0.415	0.409
[34.1–137.3]		(9)	(13)	(10)	(7)	(11)	(9)	(11)
SW (65.9)	0.446		0.167	0.478	0.260	0.429	0.352	0.366
[36.9–159.8]	(0.700)		(8)	(8)	(5)	(8)	(7)	(7)
W (64.6)	0.603	0.331		0.269	0.161	0.255	0.369	0.248
[38.4–151.2]	(0.999)	(0.841)		(9)	(5)	(10)	(12)	(9)
NW (40.5)	0.506	0.415	0.337		0.292	0.447	0.391	0.360
[24.5–103.8]	(0.999)	(0.766)	(0.929)		(7)	(8)	(7)	(6)
N (44.0)	0.536	0.402	0.236	0.428		0.490	0.463	0.474
[23.5–128.4]	(0.666)	(0.501)	(0.363)	(0.945)		(6)	(6)	(8)
NE (47.7)	0.531	0.493	0.337	0.490	0.470		0.581	0.505
[29.3–112.8]	(0.999)	(0.645)	(0.995)	(0.815)	(0.547)		(10)	(8)
E (57.1)	0.513	0.418	0.400	0.381	0.440	0.591		0.455
[28.9–166.3]	(0.697)	(0.623)	(0.971)	(0.723)	(0.517)	(0.919)		(6)
SE (42.5)	0.555	0.357	0.299	0.347	0.508	0.432	0.379	
[27.9–93.1]	(0.931)	(0.620)	(0.568)	(0.594)	(0.774)	(0.659)	(0.533)	

**Figure 3:**
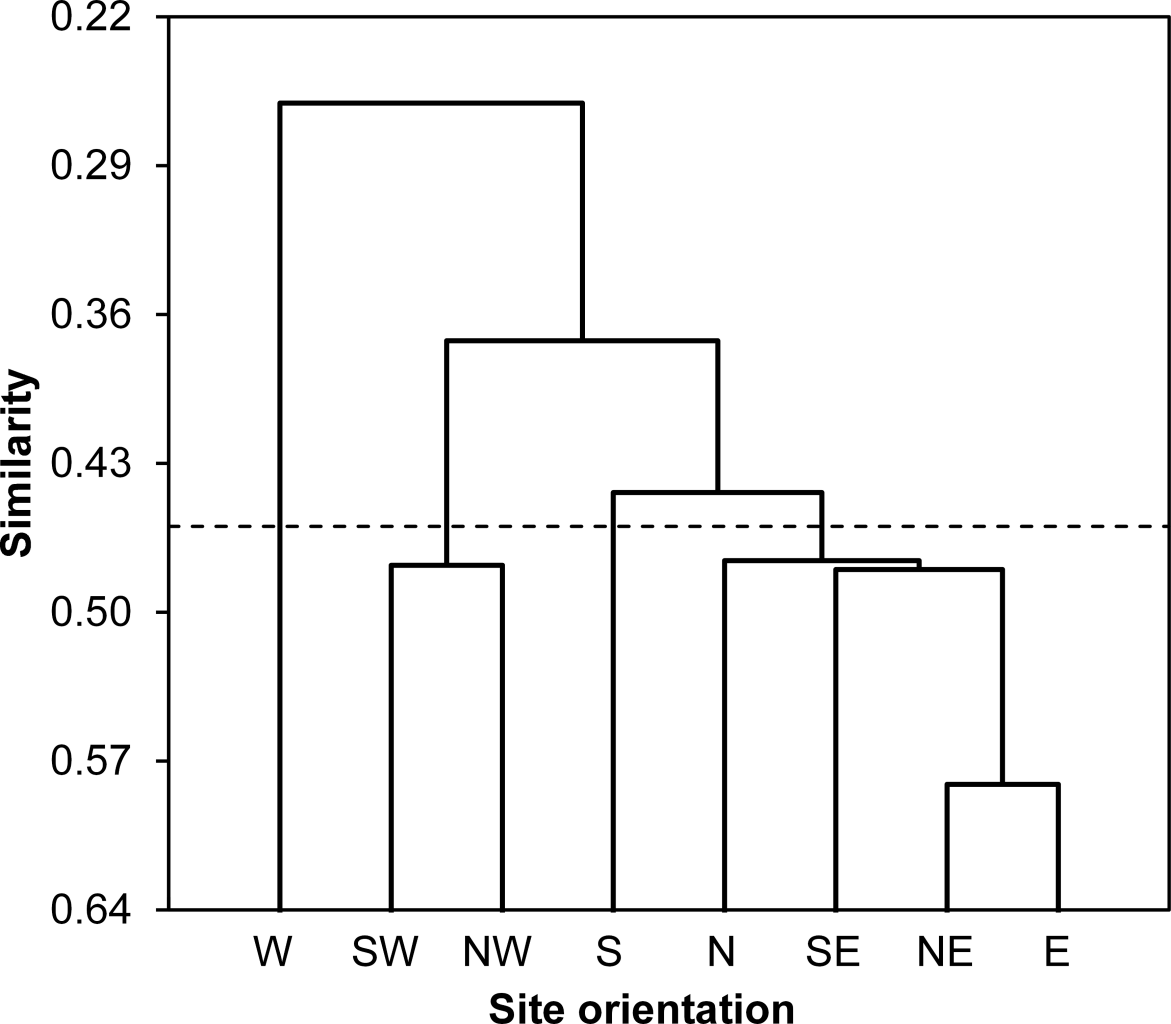
Spatial similarity clustering. Dendrogram of the agglomerative hierarchical clustering (AHC) illustrating species abundance similarity (Bray-Curtis index) among insects captured from eight stations around Sabkha Djendli (linkage rule: unweighting pair-group average).

According to values of Bray-Curtis’s index, the eight sampled stations were clustered using AHC into four different groups: (i) the first group gathered all stations located at North and East of the Sabkha including N, NE, E and SE stations, (ii) the South station was distinguished alone, (iii) the third cluster included SW and NW stations, and (iv) the fourth group was represented by West station (Fig. 3).

### Structure and diversity of functional trophic groups

Predators and saprophages held the highest catch rates with 37.3% and 26.7% of the total, respectively. Predators were more pronounced in south stations (42 ind.) especially in autumn (74 ind.) and spring (62 ind.), while saprophages are concentrated in southwest (24 ind.) and south (22 ind.) stations during the summer (46 ind.).

In terms of numbers of species, phytophages were the most abundant, with 30 species distributed almost equally along seasons and sampled stations. As for *Ni*/*S*_obs_ ratio, it varied between 1 and 11 with an average of 3.2 in study stations and seasons, i.e., that each species of a given trophic group comprises an average of 3.2 individuals. This ratio is higher in saprophages with 12.9, chiefly in stations of south (11), northwest (8.5), southwest (8), during the summer (9.2) and spring (9). Predators came in second place with 8.5 individuals per species.

The Shannon’s index showed high diversity among phytophages (*H*′ = 4.3) in both seasons and sampled stations. The values of this index were lower among predators and less important in polyphages. Regarding saprophages, their diversity values were the lowest. Similarly, evenness in coprophages (0.91), phytophages (0.87) and polyphages (0.83) showed higher values compared to values of predators (0.68) and saprophages (0.36). It is noteworthy that apart from evenness, the coprophages indicated the lowest values of ecological indices calculated for different trophic groups of insects.

The Chi-square test revealed a significant dependence for the distribution of the number of individuals of trophic groups along the orientations (*χ*^2^ = 80.62, *P* < 0.001) and seasons (*χ*^2^ = 24.57, *P* = 0.002) (Table 5). However, no significant dependence was observed for the rest of the features (Species richness, *Ni*/*S*_obs_ ratio, Shannon index, evenness) of trophic groups according to stations orientations and seasons.

**Table 5:** Spatiotemporal structure and diversity of trophic groups. Spatial and seasonal variations of insect trophic guilds living in Sabkha Djendli, Northeast Algeria.

Parameter	Orientations								Seasons			Total
	S	SW	W	NW	N	NE	E	SE	Spring	Summer	Fall	
Individual numbers	(${\chi }_{28}^{2}=80.62$, *P* < 0.00)								(${\chi }_{8}^{2}=24.57$, *P* = 0.002)			
Coprophages	4	1	4	1	0	3	3	4	8	5	7	20 (4.6%)
Phytophages	20	10	13	7	8	6	11	14	35	26	28	89 (20.5%)
Polyphages	4	3	19	7	5	4	2	3	22	13	12	47 (10.8%)
Predators	42	10	21	12	28	21	15	13	62	26	74	162 (37.3%)
Saprophages	22	24	3	17	11	16	12	11	36	46	34	116 (26.7%)
Species richness (*S*_obs_)	(${\chi }_{28}^{2}=15.65$, *P* = 0.971)								(${\chi }_{8}^{2}=4.81$, *P* = 0.777)			
Coprophages	4	1	3	1	0	2	3	3	4	4	5	7 (9.3%)
Phytophages	8	9	8	7	6	6	6	9	19	14	15	30 (40%)
Polyphages	4	3	5	4	3	2	2	2	9	5	8	10 (13.3%)
Predators	8	8	8	5	5	8	4	6	10	9	15	19 (25.3%)
Saprophages	2	3	3	2	3	4	4	2	4	5	8	9 (12%)
Ratio *Ni*/*S*_obs_	(${\chi }_{28}^{2}=22.09$, *P* = 0.779)								(${\chi }_{8}^{2}=3.35$, *P* = 0.911)			
Coprophages	1.0	1.0	1.3	1.0	0.0	1.5	1.0	1.3	2.0	1.3	1.4	2.9
Phytophages	2.5	1.1	1.6	1.0	1.3	1.0	1.8	1.6	1.8	1.9	1.9	3.0
Polyphages	1.0	1.0	3.8	1.8	1.7	2.0	1.0	1.5	2.4	2.6	1.5	4.7
Predators	5.3	1.3	2.6	2.4	5.6	2.6	3.8	2.2	6.2	2.9	4.9	8.5
Saprophages	11.0	8.0	1.0	8.5	3.7	4.0	3.0	5.5	9.0	9.2	4.3	12.9
Shannon’s index	(${\chi }_{28}^{2}=13.18$, *P* = 0.992)								(${\chi }_{8}^{2}=0.58$, *P* = 0.999)			
Coprophages	2.0	0.0	1.5	0.0	0.0	0.9	1.6	1.5	1.9	1.9	2.2	2.5
Phytophages	2.5	3.1	2.7	2.8	2.4	2.6	2.2	2.8	3.8	3.5	3.7	4.3
Polyphages	2.0	1.6	1.2	1.8	1.4	0.8	1.0	0.9	2.6	2.0	2.9	2.7
Predators	2.5	2.9	2.8	2.1	1.3	2.4	1.7	2.0	2.5	2.4	2.9	2.9
Saprophages	0.3	0.9	1.6	0.5	1.1	1.2	1.2	0.4	0.9	0.7	1.6	1.2
Evenness	(${\chi }_{28}^{2}=17.71$, *P* = 0.999)								(${\chi }_{8}^{2}=3.85$, *P* = 0.999)			
Coprophages	1.00	0.00	0.95	0.00	0.00	0.92	1.00	0.95	0.95	0.96	0.96	0.91
Phytophages	0.85	0.98	0.88	1.00	0.93	1.00	0.86	0.89	0.90	0.92	0.93	0.87
Polyphages	1.00	1.00	0.50	0.92	0.86	0.81	1.00	0.92	0.80	0.88	0.95	0.83
Predators	0.83	0.97	0.92	0.92	0.56	0.80	0.84	0.79	0.76	0.75	0.75	0.68
Saprophages	0.27	0.56	1.00	0.52	0.69	0.59	0.60	0.44	0.47	0.30	0.52	0.36

While the Chi-square test revealed a significant dependence for abundances of trophic groups among site directions and seasons (Table 5). The GLMs showed different effects of the two factors on the abundance of each trophic group. While abundances of predator species varied significantly between station orientations, seasons and their interaction (*P* < 0.001), polyphages only varied significantly between orientations whereas saprophages varied between orientations and the interaction Orientation × Season. Coprophages followed by polyphages were the insect groups less affected by seasonal and spatial variations (AIC = 278 and AIC = 1095, respectively) (Table 6).

**Table 6:** Generalized linear models. GLMs testing the variation of abundance of insect trophic guilds between seasons and station orientations in Sabkha Djendli, Northeast Algeria.

Trophic groups	Variation	*Df*	*χ* ^2^	*P*
Coprophages	Orientation	7	9.80	0.200
(AIC = 278)	Season	2	0.72	0.697
	Orientation × Season	14	17.22	0.245
Phytophages	Orientation	7	12.40	0.088
(AIC = 9120)	Season	2	1.47	0.478
	Orientation × Season	14	17.72	0.220
Polyphages	Orientation	7	26.92	<0.001
(AIC = 1095)	Season	2	3.69	0.158
	Orientation × Season	14	21.41	0.092
Predators	Orientation	7	35.29	<0.001
(AIC = 11129)	Season	2	25.76	<0.001
	Orientation × Season	14	42.73	<0.001
Saprophages	Orientation	7	24.94	<0.001
(AIC = 12124)	Season	2	2.09	0.352
	Orientation × Season	14	27.36	0.017

### Relationship between insect communities and vegetation

**Figure 4:**
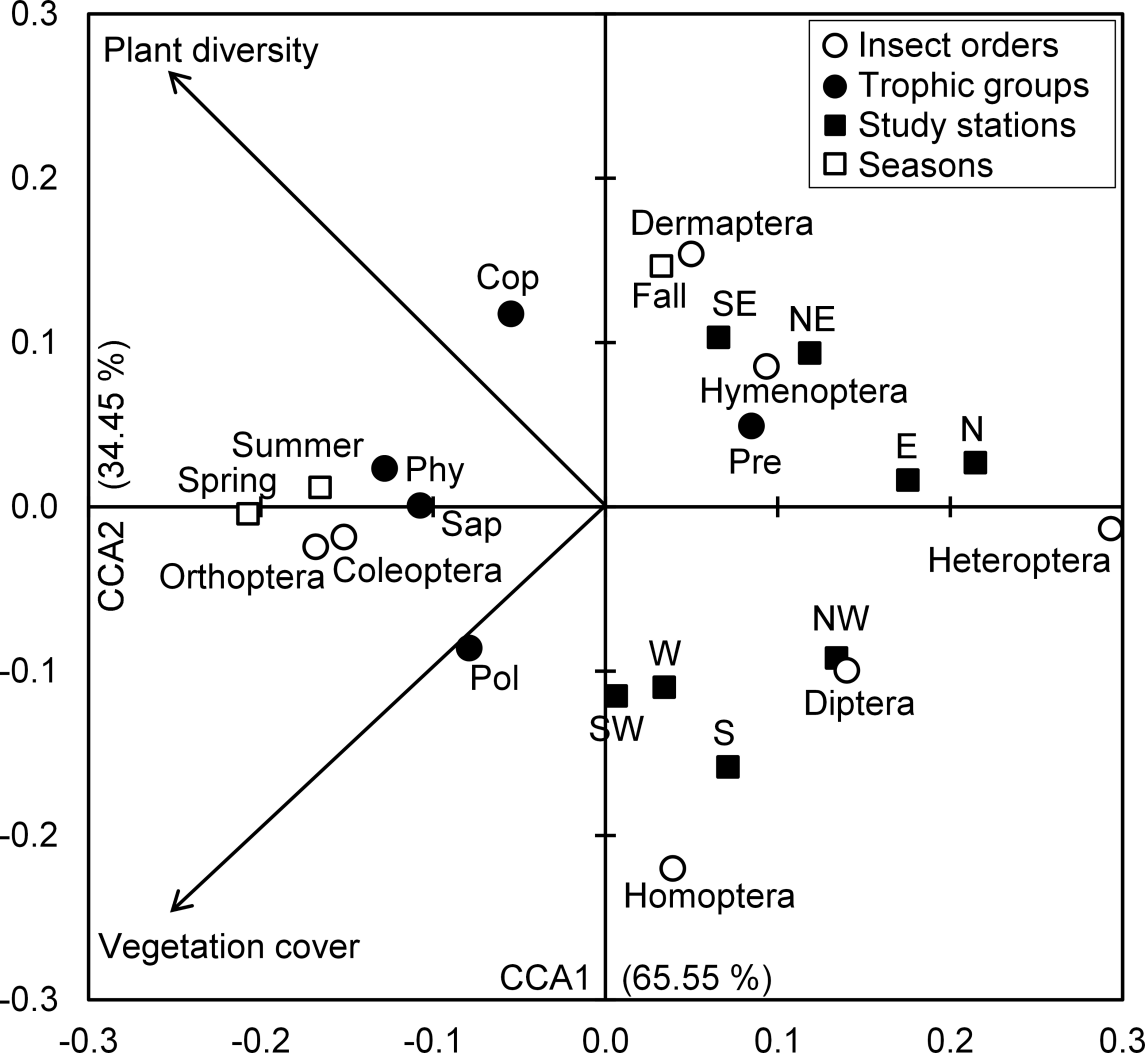
Insects-vegetation relationships. Diagram of the canonical correspondence analysis (CCA) relating spatial and seasonal densities of insect assemblages of both taxonomic orders and trophic groups with vegetation cover and species richness. (Cop, coprophages; Phy, phytophages; Pol, polyphages; Pre, predators; Sap, saprophages).

The Eigenvalues of CCA applied for insect assemblages and vegetation parameters in canonical axis 1 and 2 were high and explained 65.55% and 34.45% of constrained inertia, respectively. According to CCA, the density of polyphages was positively associated with vegetation cover, but this parameter had a negative influence on the number of individuals of predators, Hymenoptera and Dermaptera, especially in autumn at northeast, southeast, north and east stations. In addition, coprophage abundance was positively related with species richness of plants; however, Diptera and Homoptera were located on the negative side of the axis representing species richness of plants, and this in northwest, west, southwest and west stations. The phytophages and saprophages as well as Coleoptera and Orthoptera were also positively correlated with both axes of vegetation parameters, particularly in spring and summer seasons. Conversely, the two parameters of vegetation negatively influenced Heteroptera densities in north and east stations (Fig. 4).

All the significant correlations we obtained were positive with both vegetation parameters. These concerned the abundances of Dermaptera in relation with species richness of plants, and Orthoptera and Coleoptera with vegetation cover and richness of plants. As for the trophic groups of the entomofauna, the correlation test was significant for the numbers of polyphages, phytophages and saprophages vs. vegetation cover on the one hand, and coprophages, phytophages and saprophages vs. plant species richness on the other hand. Moreover, the correlation tests revealed a significant decrease in the abundances of Hymenoptera with the increase of precipitation (*r* = − 0.080, *P* < 0.05), but a positive relationship was observed between predator abundances and wind speed (*r* = 0.206, *P* < 0.05). The rest of climates parameters were not significantly linked to taxonomic orders and trophic groups (Table 7).

**Table 7:** Insects-ecosystem correlations. Pearson correlation tests between abundance of orders and trophic guilds of insects and climate parameters (T, average of mean temperatures; PP, total of precipitation; HM, average of mean humidity; WS, mean wind speed; RA, number of rain days) and spontaneous vegetation characteristics (cover and species richness) of Sabkha Djendli, Northeast Algeria.

Variables	Climate parameters					Vegetation parameters	
	T (°C)	PP (mm)	HM (%)	WS (Km/h)	RA (days)	Vegetation cover	Species richness
**Taxonomic orders**							
Dermaptera	0.076	0.089	−0.090	0.021	0.086	0.214	0.613^*^
Orthoptera	−0.007	0.027	−0.062	0.093	0.018	0.717^*^	0.796^**^
Heteroptera	0.022	−0.082	0.188	−0.283	−0.056	−0.030	0.109
Homoptera	−0.266	−0.205	0.103	0.214	−0.223	0.591	0.295
Coleoptera	−0.026	−0.020	0.010	0.021	−0.022	0.656^*^	0.742^**^
Hymenoptera	−0.056	−0.080^*^	0.095	−0.052	−0.075	0.215	0.505
Diptera	−0.076	−0.126	0.164	−0.121	−0.115	0.308	0.257
**Trophic groups**							
Coprophages	0.038	0.087	−0.128	0.122	0.075	0.429	0.786^**^
Phytophages	0.054	0.070	−0.078	0.034	0.067	0.586^*^	0.748^**^
Polyphages	0.123	0.140	−0.137	0.020	0.137	0.604^*^	0.571
Predators	−0.065	0.008	−0.090	0.206^*^	−0.011	0.262	0.488
Saprophages	0.020	−0.011	0.044	−0.085	−0.003	0.591^*^	0.717^*^

**Notes.**

* Significant correlations for *P* < 0.05.

** Significant correlations for *P* < 0.01.

## Discussion and Conclusion

Salt lakes offer exceptional conditions for ecological studies of aquatic ecosystems, due to the frequency and intensity of changes in the biological communities compared to freshwater ecosystems (Comin & Comin, 1992). This feature is most notable in arid regions, so that these habitats are home to many original and well-adapted life forms (Chenchouni, 2012a).

Out of all the conducted samples, the Sabkha of Djendli houses 75 insect species related to 31 families and 7 orders. In terms of individual numbers caught, the orders of Coleoptera and Hymenoptera dominate other insect orders, while Dermaptera, Heteroptera, Homoptera and Diptera are slightly present with very similar densities in different study stations. This distribution of the composition could be attributed to the low dispersal ability of these insects, as well as the scarcity of these categories (Cobos, 1987), but mostly to the ineffectiveness of pitfall traps to capture flying insects since this type of trap is specifically designed for ground arthropods (Spence & Niemelä, 1994). The interpolated species accumulation curves based individual-rarefaction highlighted the key contribution of rare species ‘singletons’, and secondary doubletons, for estimating the number expected species.

Since saline environments in hot arid regions are characterized by large spatial and temporal fluctuation of water level and salinity, community of inland insects can be modelled either by the synergistic effect of several factors (abiotic and biotic) that are related to these two parameters; or by the predominance of one factor over others (e.g., vegetation parameters) (Vidal-Abarca, Gómez & Suárez, 2004; Velasco et al., 2006). Moreover, the state of the composition of insect assemblage in inland saline environments can be explained by the morphological and physiological adaptations necessary to cope with the extreme and unpredictable conditions of these habitats on one hand, and their life cycle, phenological adaptations and behaviour on the other hand (Cloudsley-Thompson, 1975; Louw & Seely, 1982).

The study of variations in the frequency of abundance and occurrence of different insect orders shows that the beetles represent the most abundant order that appears regularly in different sampling stations of Sabkha Djendli and during the study period. This frequency is reflected by the presence of three constant species (Coleoptera and Hymenoptera), six common species (Coleoptera, Hymenoptera and Dermaptera) and 66 accidental species. This finding is in contrast to the observation made by Boix et al. (2008) where it has been found that beetles are the most affected group within insects of saline environments, while our results are similar to those of Vidal-Abarca, Gómez & Suárez (2004) who argue that in the salt wetlands of arid and semi-arid areas, Coleoptera and Diptera were the most abundant groups because of their large adaptation to critical and extreme conditions. It is well known that the beetles are the most abundant and occurring insect group in nature (Bouchard et al., 2011). In addition to their dominance in the animal kingdom, they are an important food resource for consumers at different levels in the food web; their number of species represents a good biological indicator of habitat quality (Rainio & Niemelä, 2003; Sánchez-Fernández et al., 2006). Moreover, because of their sensitivity to environmental modifications, they constitute a model of choice for assessing the diversity of habitats (Haddad et al., 2009).

Regarding insect species richness, the highest value is recorded in the west and south stations with 27 species. According to Neffar, Chenchouni & Si Bachir (in press), these stations are characterized by certain homogeneity in their floristic composition. These areas are grazed and fertilized by dung they receive and therefore stimulate the development of certain flowering herbaceous and thus attract more pollinators. While cattle dung favor the abundance of coprophages, mostly Scarabaeidae in our case. These observations were confirmed by the CCA where we found that coprophages density was positively correlated with plant diversity, which was negatively associated with west and south stations. The vegetation significantly affects the different trophic groups (herbivores, parasitoids and predators) of the insect fauna living at the herbaceous layer, through its floristic composition and functional diversity (Koricheva et al., 2000), but also through the density of vegetation cover that creates a microclimate for soil-dwelling species (Siemann, 1998). According to Haddad et al. (2009), species richness of predators and herbivores is positively related to species richness and plant biomass, without being affected by its composition. However, in lentic ecosystems, high electrical conductivity causes a significant decline in the abundance and taxonomic richness of macroinvertebrate fauna (Waterkeyn et al., 2008; Carver et al., 2009).

Based on the values of the Shannon index and evenness, insect diversification is well marked in the different stations and seasons, indicating a balance between the number of sampled invertebrate populations, although it may be that the constituent species of assemblages are generalists, adapting to most environmental conditions, as suggested by Rainio & Niemelä (2003) and Montagna et al. (2012).

Furthermore, the dominance of accidental species (66/75) may be connected to the sparse structure of vegetation of the Sabkha. Because the presence of dense vegetation reduces predation against herbivores that therein also find abundant food, but also reduces the antagonistic effect between predators (Finke & Denno, 2002); this is not the case with the open vegetation of Sabkha Djendli, which is characterized by a medium to low coverage (Neffar, Chenchouni & Si Bachir, in press). Otherwise the same type of structure and composition of vegetation cover are almost noted in arid and semi-arid wetlands of Algeria and North Africa (Khaznadar, Vogiatzakis & Griffiths, 2009; Chenchouni, 2012b). This particular pattern of species occurrences in Sabkha Djendli may also be explained by the unpredictable environmental changes inciting species to the coexistence, and consequently the increase of diversity (Chesson & Huntly, 1997; Piñero et al., 2011). But generally, seasonality remains the primary determinant factor of invertebrate diversity in any ecosystem (Wolda, 1988). The metabolism of poikilotherms requires low investment in energy, making these invertebrates highly effective organisms for the survival in extreme environments (Heatwole, 1996). This explains the significant variation in predator numbers between the studied seasons and stations. This may be related to climate factors, mainly precipitations and wind speed, which both showed significant correlations with numbers of predators and hymenoptera. As most of hymenopteran species being predators (except species belonging to Apidae, Megachilidae and Halictidae) and have their populations increase in late spring and during the hot season ‘summer,’ the precipitation revealed negative correlation because it occurs mostly in autumn and moderately in the spring.

The study of trophic status of insect species reveals their affiliation to different ranks of consumers and thus these species virtually occupy different levels in the food web. Species richness decreases in the following order herbivores> predators> polyphages> saprophages> coprophages with 40%, 25.3%, 13.3%, 12.0% and 9.3%, respectively. According Piñero et al. (2011), seasonal variations have profound effects not only on the number of species, abundance and biomass of invertebrates during different times of the year, but also on the trophic and functional structures of communities. For his part, Siemann (1998) suggested that the diversity, quality and/or composition of plant species can in their turn influence the diversity of higher trophic levels, not only by changing the diversity of herbivores, parasites and predators but also by affecting the quality of the food of herbivores and the ease with which they can be captured. Therefore, the spatiotemporal variation in traits of vegetation (composition and cover) between the eight stations and seasons (Neffar, Chenchouni & Si Bachir, in press) is the cause of the significantly uneven spatiotemporal distribution (according to Chi-square test) of insect group densities. Indeed, the CCA has allowed the characterization of insect assemblage responses to vegetation parameters.

The comparison of specific composition between different stations of Sabkha Djendli using the Jaccard index shows low similarity values, commonly not exceeding 35%. This similarity would find its explanation in the heterogeneity of ecological conditions for this fauna, in particular the composition and structure of the sparse vegetation which is chiefly composed of halophytes including *Suaeda* spp. *Atriplex* spp. and *Salicornia* spp. (Neffar, Chenchouni & Si Bachir, in press), reflecting thus the degraded conditions prevailing on the physicochemical properties of soil in which they grow (Khaznadar, Vogiatzakis & Griffiths, 2009). According to Baguette (1992), the inter- and intra-specific competitions, predation and parasitism regulate the spatial and temporal distribution of species and structure of communities. Also, the distribution of a given species is a dynamic phenomenon that involves a set of extinction and recolonisation stages of local populations following changes in environmental conditions. Even more so, several studies have shown that changes in communities across habitats are influenced by environmental variables, in particular the type of substrate (Ligeiro, Melo & Callisto, 2010) and even the coarse organic matter (Hepp & Melo, 2013).

Furthermore, we speculate that the land use around each station has an important influence on the variation of insect abundance and composition in that station. The AHC grouped together N, SE, NE and E stations, and these are furthest to the urban area and cropping lands, while other groups of stations (S, SW, W, NW) are the closest from urban and crop areas. Similarly, N and NW stations are clustered together because both are closest to montane forests and furthest from urban areas. The land use is an important factor that helps to identify spatiotemporal variation in insect communities (Schweiger et al., 2005). Human activities in general and the agricultural ones in particular, including grazing and animal farming, may induce several changes to structural and organisational features of insect populations and communities (Rand, Tylianakis & Tscharntke, 2006).

The spatial variability of the insect fauna of Sabkha Djendli is related to the combination of several factors, among others: the climate is critical to the distribution of arid arthropods (Langlands, Brenna & Pearson, 2006), the reproductive potential and dispersal capabilities (Thompson & Townsend, 2006), and environmental heterogeneity may be a contributing factor to their low dispersion.

The halophytic belt of Sabkha Djendli have a high richness of insects especially in spring and autumn, coinciding in part with their breeding period. As the recorded species are mostly phytophages, their number naturally increases in the spring with the increase of plant diversity and vegetation cover, whereas the predators generally depend on the availability of prey (Koricheva et al., 2000; Haddad et al., 2009). This statement is supported by findings of the CCA where the abundant insect groups (Coleoptera, phytophages) were found linked to vegetation parameters mainly in spring and summer.

Following this study, the use of pitfall traps in Sabkha Djendli revealed some knowledge about the entomofauna. The insect community shows high taxonomic richness and diversity in different stations and seasons. The composition of functional trophic groups are closely related to diversity and vegetation cover. The conservation of this biological heritage, so rich but little known, non-invested and generally underestimated by managers, can only be possible by improving and deepening our knowledge about biodiversity including the functional communities in relation with threatening factors and disturbances that affect their vital activities.

## Supplemental Information

10.7717/peerj.860/supp-1 Table S1 Raw count-data of insect species Taxonomix list of insect species with their number of individuals trapped during three seasons (2006) and at eight stations around Sabkha Djendli (northeast Algeria).

10.7717/peerj.860/supp-2 Table S2 Total abundances of insect species Model of total raw data (multiple individual-based abundance samples) utilized for species richness estimation using the program EstimateS 9.1.0.

10.7717/peerj.860/supp-3 Table S3 Raw input data at station scale Model of raw data used for the analysis of shared species estimators and indices using EstimateS 9.1.0 based insect species abundances recorded at the eight sampled stations of Sabkha Djendli.

10.7717/peerj.860/supp-4 Table S4 Daily climatic data of 2006 Daily mean temperatures, precipitation, mean humidity, mean wind speed, and indicator for occurrence of rain in the Sabkha of Djendli (Batna, northeast Algeria) during 2006.

10.7717/peerj.860/supp-5 Table S5 Statistics of estimated species richness and diversity Diversity statistics (accumulated species and individuals, richness estimators, species diversity indices) computed by EstimateS 9 based on total captures of insects in Sabkha Djendli (northeast Algeria).

10.7717/peerj.860/supp-6 Table S6 Shared species statistics Shared species statistics computed by EstimateS 9 forsample-based abundance data of insects trapped at eight stations of Sabkha Djendli (northeast Algeria).
